# Impact of Infectious Disease after *Lactococcus lactis* Strain Plasma Intake in Vietnamese Schoolchildren: A Randomized, Placebo-Controlled, Double-Blind Study

**DOI:** 10.3390/nu14030552

**Published:** 2022-01-27

**Authors:** Nghiem Nguyet Thu, Truong Tuyet Mai, Tran Thị Thu Trang, Nguyen Anh Tuan, Tran Chau Quyen, Nguyen Lien Hanh, Nguyen Huu Hoan, Bui Thi Huong Lan, Phung Thi Hau, Ha Huy Tue, Truong Viet Dung, Ryohei Tsuji, Yuta Watanabe, Naoki Yamamoto, Osamu Kanauchi

**Affiliations:** 1 National Institute of Nutrition, 48B Tang Bat Ho, Hanoi 100000, Vietnam; nghiemnguyetthu@dinhduong.org.vn (N.N.T.); tranthithutrang@dinhduong.org.vn (T.T.T.T.); nguyenanhtuan@dinhduong.org.vn (N.A.T.); tranchauquyen@dinhduong.org.vn (T.C.Q.); nguyenlienhanh@dinhduong.org.vn (N.L.H.); nguyenhuuhoan@dinhduong.org.vn (N.H.H.); buithihuonglan@dinhduong.org.vn (B.T.H.L.); phamthihau@dinhduong.org.vn (P.T.H.); hahuytue@dinhduong.org.vn (H.H.T.); 2 Hanoi Medical University, 1 Ton That Tung, Dong Da, Hanoi 100000, Vietnam; dzungtruong53.hmu@gmail.com; 3 Kirin Central Research Institute, Kirin Holdings Co., Ltd., 1-13-5 Fukuura Kanazawa-ku, Kanagawa, Yokohama-shi 236-0004, Japan; Ryohei_Tsuji@kirin.co.jp; 4 Research and Development Strategy Department, Kirin Holdings Co., Ltd., 4-10-2 Nakano, Nakano-ku, Tokyo 164-0001, Japan; Yuta_Watanabe@kirin.co.jp (Y.W.); kanauchio@wonderfarmonline.com (O.K.); 5 Genome Medical Sciences Project, National Center for Global Health and Medicine, 1-7-1 Kohnodai, Ichikawa-shi, Chiba 272-8516, Japan; nyamamoto0508@gmail.com; 6 Tokyo Medical and Dental University, 1-5-45 Yushima, Bunkyo-ku, Tokyo 113-8519, Japan; 7 Interfood Shareholding Company, 127 Lo Duc, Hanoi 100000, Vietnam

**Keywords:** *Lactococcus lactis* strain Plasma, plasmacytoid dendritic cells, infectious disease

## Abstract

*Lactococcus lactis* strain Plasma (LC-Plasma) is reported to have anti-viral effects via direct activation of plasmacytoid dendritic cells, which upregulate the production of type I and III interferons. A randomized, placebo-controlled, double-blind, parallel group study was designed for elementary schoolchildren, grades 1 to 3, in Vietnam. LC-Plasma or a control were administered to schoolchildren as a beverage (1.0 × 10^11^ count LC-Plasma/day/person). The primary endpoint was to determine the efficacy of LC-Plasma in reducing the cumulative days absent from school due to upper respiratory disease (URID) and gastrointestinal disease (GID), and the secondary endpoint was to evaluate the potency of LC-Plasma on URID/GID symptoms and general well-being scores. LC-Plasma intake significantly reduced the cumulative days absent from school due to URID/GID (Odds ratio (OR) = 0.57, *p* = 0.004) and URID alone (OR = 0.56, *p* = 0.005); LC-Plasma also significantly reduced the number of cumulative fever positive days during the first 4 weeks of intervention (OR = 0.58, *p* = 0.001) and cumulative days with diarrhea during the last 4 weeks of the intervention period (OR = 0.78, *p* = 0.01). The number of positive general wellbeing days was significantly improved in the LC-Plasma group compared with the control throughout the intervention period (OR = 0.93, 0.93, *p* = 0.03, 0.04 in the first and last 4 weeks of the intervention, respectively). These data suggest that LC-Plasma seems to improve the health condition of elementary schoolchildren and reduces school absenteeism due to infectious disease, especially URID.

## 1. Introduction

Changes in human ecology, such as global warming and an increased geographical movement of people and goods, have dramatically increased the risk of viral infection; however, the efficacy of vaccines and remedies for infectious diseases is limited by the high mutation rates of viruses [1]. Influenza is a malady of the human respiratory system, which causes economic and social burdens on society, especially among children and the elderly. The cost of children under 14 years old with influenza-like illness (ILI) was reported to be about 140 USD/episode and this burden is a serious issue in Vietnam [2]. In 2018, an ILI sentinel surveillance in northern Vietnam reported that 13.4% of ILI patients were influenza virus positive and the dominant pathogens were A/H3N2 and B [3]. It was also reported that the highest morbidity rate was observed from June to September. In addition, Thi et al. reported that the highest proportion of influenza was in the 5-to-14 age group during 2006 to 2013 surveillance in Vietnam [4].

Acute gastroenteritis, caused by norovirus and rotavirus, is a major and critical disease in young children, and it is estimated that 5300–6800 children die annually from this disease in Vietnam [5]. These acute gastrointestinal infectious diseases also cause high economic and social burdens in Vietnam [6]. Prevention of infectious disease incidence in children is very important, in view of, not only health care and public health, but also socio-economic issues. The nutritional status of children is an important factor in health care and acts as a predictive indicator for infectious diseases in Vietnam, where malnutrition is a major problem [7,8]; therefore, we have paid particular attention to weight, height, and age parameters, and the Z-score. The Z score is a statistical measure that reflects the relative deviance from the median value, and is comparative data. The Z score is widely used in nutritional evaluation as a reference frame by many organizations and scientists.

Lactic acid bacteria (LAB) belong to a large group of rod- or cocci-shaped Gram-positive bacteria, commonly used in the production of food and probiotic supplements. Ingestion of LAB can aid in lactose digestion, prevent and treat diarrheal diseases, and enhance the immune system of the digestive tract [9,10]. Several probiotics and paraprobiotics (sterilized probiotics) were reported to be effective in preventing and treating virally-induced gastrointestinal and respiratory infectious diseases [11]. Although the detailed mechanism is still under investigation, it is considered that probiotics and paraprobiotics play an important role in modulating the cross-talk between commensal bacteria and the mucosal immune system [12].

*Lactococcus lactis* strain Plasma (LC-Plasma) is a synonym of *Lactococcus lactis* subsp. *lactis* JCM 5805. LC-Plasma was recently shown to directly activate plasmacytoid dendritic cells (pDCs), which in turn up-regulates production of type I and III interferons (IFN)s [13]. Oral LC-Plasma administration to animal models improved symptoms caused by viral infection in gastrointestinal (rotavirus) and respiratory (parainfluenza) diseases [14,15]. A previous clinical trial suggested that oral supplementation of LC-Plasma clearly prevented the pathogenesis of influenza-like illnesses through up-regulation of IFN-α mediated responses [16]. Furthermore, a community-based intervention study examined the effect of consumption of a yoghurt containing LC-Plasma on influenza incidence rates among schoolchildren in Japan: LC-Plasma reduced both the incidence rate and cumulative incidence rate of influenza [17]. No safety concerns were reported for the intake of heat-killed LC-Plasma, either in the long-term or excessive intake of beverage and capsule forms of LC-Plasma [18,19]. We hypothesized that oral supplementation of LC-Plasma in Vietnamese schoolchildren may prevent the incidence of infectious diseases, which would reduce the number of school absent days.

The primary endpoint of this study was to determine the efficacy of LC-Plasma in reducing the cumulative days absent from school due to infectious disease, including upper respiratory infectious disease (URID) and gastrointestinal infectious disease (GID), and to assess the impact of LC-Plasma in improving the severities of URID or GID-like symptoms in healthy Vietnamese elementary schoolchildren.

## 2. Materials and Methods

### 2.1. Statement of Ethics

The trial protocol and all amendments were reviewed and approved by the Institutional Review Board at the National Institute of Nutrition (NIN) (No. 370/VDD-QLKH) and the Scientific Committee at the NIN (No. 1305/QD-VDD). All procedures involving human participants were in accordance with the ethical standards of the institutional and/or national research committee and with the 1964 Helsinki Declaration, and its later amendments or comparable ethical standards. This study was registered at the University Hospital Medical Information Network Clinical Trial Registry as UMIN000041843.

### 2.2. Study Design

This study was designed as a randomized, placebo-controlled, double-blind clinical trial. Approximately 1000 healthy Vietnamese children, from grades 1 to 3 in elementary schools in Ninh Binh province, were recruited for this non-invasive, intervention study. Four schools were selected as follows: Yen Lam Primary School from Yen Lam Commune, Yen Thai Primary School from Yen Thai Commune, Pham Nhat Duat Primary School from Yen Mac Commune, and Khanh Duong Primary School from Khanh Duong Commune. 

### 2.3. Enrollment and Selection of Participants

School students were invited to the recruitment process, where schoolchildren and their guardians were given information about the trial and asked to participate. Guardians provided written informed consent, and completed the preliminary questionnaires for their participating children. The rationale of the study size was determined by G*POWER (Ver. 3.1) (calculation of effect size 0.1, 5% precision and a 95% confidence interval), and it was estimated that 442 participants would be required per group; 10% of the recruited participants were expected to withdraw from the study through non-compliance, developing underlying diseases, or other reasons. Eligible participants were enrolled into the study, based on the inclusion and exclusion criteria. At the monitoring meeting at enrollment, data on body height, weight, general wellbeing, and basic information were recorded by a participant’s guardians in an enrollment information sheet, and these data were used as background information for this study. The eligible participants were divided into two groups (Group A and B), to have no bias in gender, age, and weight, with the person in charge of data analysis of this study using the random number table. 

Participants were immediately divided into two groups during the pre-intervention period (T1), based on age, gender, and school.

### 2.4. Inclusion and Exclusion Criteria

Inclusion criteria:

(1) Signed and dated informed consent form provided, after willingness of participants (guardians) confirmed;
(2) Willingness to comply with all study procedures for the period of the study (both participants and guardians);
(3) Aged 6 to 9 years old (elementary school grades 1 to 3);
(4) Residing in Yen Mo District, Ninh Binh Province, with Vietnamese citizenship (both participants and guardians);
(5) No underlying chronic sickness from respiratory disease and/or gastrointestinal disease at enrollment (participants).

Exclusion criteria:

(1) Unable to provide signed and dated informed consent form (guardian);
(2) Unable to comply with all the study procedures (participants and guardians);
(3) Allergic to milk (contained within the study products) (participants);
(4) Regularly using steroid drugs (immune suppressive drugs) (participants);
(5) Those with serious respiratory and/or gastrointestinal disease (participants);
(6) Any persons deemed inappropriate as participants by the principal investigators of this study;
(7) Those currently participating in other clinical trials (participants) or those who are planning to participate in other clinical trials during the study period (participants).

### 2.5. Pre-Intervention Period (Term 1; T1)

Eligible participants (including guardians) were each given a daily record booklet, and an explanation of their responsibilities over the trial period. Participants recorded their daily health condition in the record booklet. The detailed basic health condition of participants was monitored during T1. The intervention period was started after T1. 

### 2.6. Intervention Period (Term 2; First 4 Weeks as T2, Term 3; Latter 4 Weeks as T3) and Post-Intervention Period (Term 4; T4)

**Figure 1:**
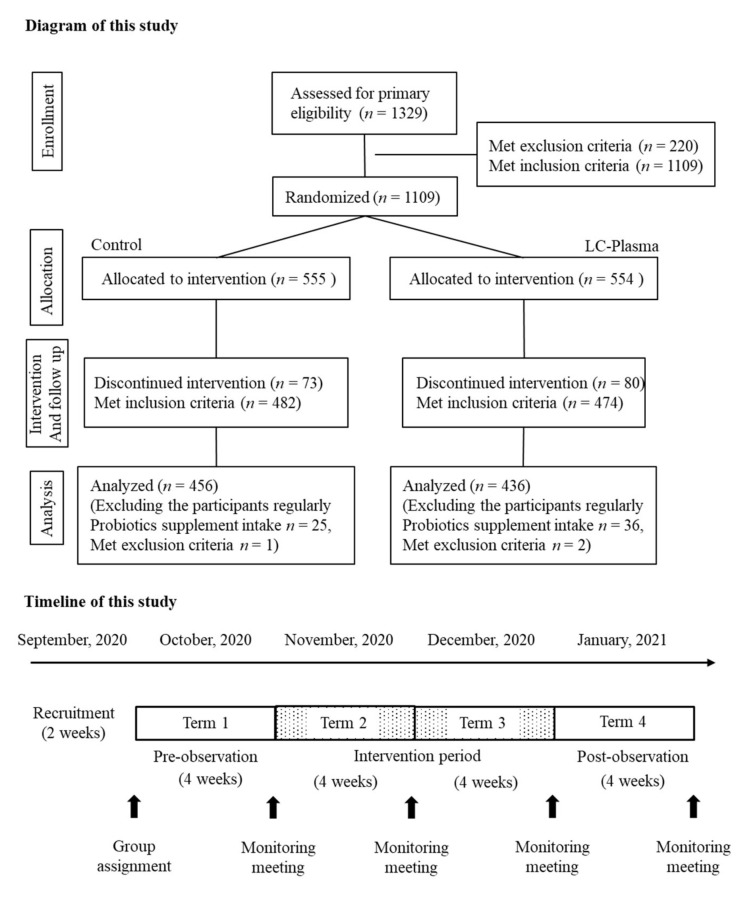
The flow of participants through this study and the timeline.

During T2 to T4, participants (guardians) recorded their health condition and ingestion of study products in the daily record booklet, and attended the monitoring meeting at the end of each term. Monitoring staff measured participants’ body weight, height, and general condition at the meetings. Eligible participants were given the study products to be consumed over 8 weeks. Physical examinations were completed at 4-week intervals. The timeline of the study is shown in Figure 1.

### 2.7. Data Collection

During T1 through T4, participants recorded date of birth, gender, school grade, medical history (positive or negative chronic disease, acute disease, any other previous diseases), allergies to milk or milk products, any use of steroids, body weight and height, and regular nutritional information in the enrollment form. Each guardian of a participant also monitored their body temperature (0; <37.5 degree Celsius, 1; ≥37.5 degree Celsius), cough score, runny nose score, constipation score, diarrhea score, abdominal pain score, general wellbeing score, medical drug usage.

### 2.8. Study Products

It has been reported that LC-Plasma affects pDC activation and reinforces responses against inactivated influenza virus in PBMC, at doses of approximately 1.0 × 10^11^ count LC-Plasma/day/person, in viable and heat-killed forms [16,20]; therefore, the daily dose level of LC-Plasma was set as 1.0 × 10^11^ count LC-Plasma/one serving size in this study. 

Study products were provided as PET-bottled beverages (280 mL/one serving size) containing sugar (5%), sweetener (0.01%), citric acid (0.04%), flavors (0.14%), skim milk powder (0.1%), and either 50 mg (more than 1.0 × 10^11^ counts) of heat killed LC-Plasma (active product for LC-Plasma group) or 50 mg maltodextrin (placebo product for control group). Both study products were produced using a beverage manufacturing process under the ISO22000/HACCP safety certificate by Interfood Shareholding Company (IFS) in Vietnam. The production manager of IFS made an allocation list of study products (Placebo or LC-Plasma) and group (A or B), then the administrative manager of IFS, who were independent from this study, sealed it and kept under blinded conditions until code breaking.

### 2.9. Definition of Score

Fever, cough, and runny nose were selected as representative symptoms of URID, according to a previous report on children’s URID symptoms in Vietnam [21]. Bowel movement malfunction occurs in GID, thus constipation, diarrhea, and abdominal pain were selected as symptoms of GID. The scale had 4 grades of severity (0—none, 1—slight, 2—moderate, 3—severe), with a modified Wisconsin scale for easy recording [22]. The general wellbeing scale was set using 5 ranks (0—good, 1—slightly good, 2—normal, 3—slightly bad, 4—bad), as described in a previous report [23]. 

### 2.10. Statistical Analysis

For parametric data, Student’s *t*-test was performed to determine if any of the parameters significantly differed between groups. Statistical analyses were performed using R software (ver. 3.6.2) and STATA (ver. 12). WHO Anthro plus Software was used to evaluate the nutritional status of children by calculating the Z-score for weight/age, height/age, and weight/height/age of participants. Gender, cumulative absent days, cumulative days with symptoms, and cumulative days of general wellbeing were analyzed by *Chi*-square test. When *Chi*-square test was used (except for gender analysis), odds ratio (OR) and its 95% of confidence interval (95% CI) were shown in Tables. Non parametric data (the intensity of score analysis and intensity of general wellbeing, body weight and height, and age) were analyzed by Wilcoxon rank sum test. It was considered that *p* values less than 0.05 were significantly different, and *p* values less than 0.1 as showed a tendency towards significance.

## 3. Results

### 3.1. Participant Characteristics and Background Data Analysis

A total of 1329 school students were screened at the trial sites and, based on the participant inclusion and exclusion criteria, 1109 volunteer schoolchildren (guardians) provided written informed consent, received the study products, and enrolled onto the study. At the end of T4, a total of 956 participants had completed the 8-week intervention, while 153 withdrew from the study. To remove the bias of probiotics, participants with regular probiotic supplement consumption were removed (control group; 25, LC-Plasma group; 36). Three participants were not eligible for this study and were not included in the analysis due to gastrointestinal dysfunction (LC-Plasma; 1) and severe upper respiratory disease (control; 1, LC-Plasma; 1) based on T1 data. The final number of participants analyzed were 456 in the control group and 436 in the LC-Plasma group (Figure 1).

The baseline characteristics of the participants, age, gender, weight, height, and Z-score, revealed no significant differences between the groups at enrollment (Table 1). There were no significant differences in body weight and height between the two groups throughout the intervention period (T2 and T3), as shown in Supplementary Table S1. In addition, changes in Z-scores for weight/age, height/age, and body mass index between groups during T2 and T3 were not significantly different between the groups (Table S1) and there were no significant differences between groups in change of respective scores during these terms (Term 1 to Term 2, Term 2 to Term 3, and Term 1 to Term 3; data not shown). The compliance of participants taking the study products during the intervention period was also monitored.

### 3.2. Analysis of Cumulative Days Absent from School

**Figure 2:**
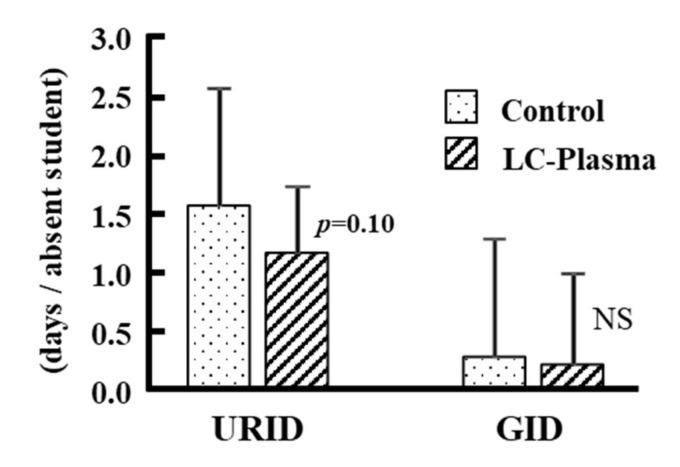
Number of absent days per absent student. URID represents upper respiratory infectious disease, and GID represents gastrointestinal infectious disease. NS; not significant difference between groups.

To determine whether there is a statistically significant difference between the expected frequencies for school absent days between groups, *Chi*-square test was used for cumulative days analysis. Table 2 shows the cumulative days absent from school. During T2, the cumulative days absent from school due to URID or GID were 70 in the control group and 38 in the LC-Plasma group, and due to URID only were 62 in the control group and 33 in the LC-Plasma group. Both results showed a significant difference between the groups (*p* < 0.01). There were no significant differences between the cumulative days absent from school due to GID only between the two groups during T2 and T3. In addition, there were no significant differences between the cumulative days absent from school due to URID or GID, URID only, and GID only between the two groups during T3. There was a tendency towards a decrease in the cumulative number of absent days due to URID or GID during the total intervention period (T2 + T3) in the LC-Plasma group (*p* = 0.063): 99 in the control group and 71 in the LC-Plasma group. The number of students absent from school during T2 and T3 due to URID or GID did not significantly differ between the groups (data not shown). In addition, the number of school absent days per absent student in T2 + T3 was determined. As shown in Figure 2, LC-Plasma intake reduced the number of absent days due to URID per student, although the difference was not significant (*p* = 0.102). Only a small number of participants were absent due to GID in this study, making it difficult to evaluate the effects of LC-Plasma intake on the number of days absent due to GID.

The number of absent days in each school per absent participant is shown as the mean and SD. The number of absent days due to URID tended to decrease in the LC-Plasma group (*p* = 0.102). During T2 and T3, the number of participants who were absent from school due to URID and/or GID was 54 in the control group and 52 in the LC-Plasma group (NS; not significant).

### 3.3. Disease Score Analysis and Cumulative Days with or without Symptoms

The sum of asymptomatic days (score; 0), and the sum of symptomatic days (score; 1, 2 or 3) for each participant during T2 and T3 are shown as the cumulative days with positive or negative URID related symptoms and GID related symptoms (Table 3). LC-Plasma intake significantly attenuated the cumulative fever positive days during T2. LC-Plasma also significantly reduced the cumulative days with diarrhea during T3. The results for cumulative days with constipation symptoms were controversial: it was significantly lower in the control group during T2 and there was a tendency to be lower in the LC-Plasma group during T3. The cumulative days with abdominal pain symptoms also tended to be lower in the LC-Plasma group during T3 (*p* = 0.084).

The intensity of GID related symptoms score (constipation score, diarrhea score, and abdominal pain score) and URID related symptoms score (fever score, cough score, and runny nose score) are shown in Supplementary Table S2. Although the fever score was slightly lower in the LC-Plasma group during T2 when compared with the control group, there were no significant differences in the intensity of all symptoms between groups during T2 and T3.

### 3.4. General Wellbeing

The intensity of general wellbeing scores during the intervention periods did not significantly differ between the two groups, although the mean value in the LC-Plasma group was slightly lower than that in the control group (Supplementary Table S3). The general wellbeing scores were cumulated and divided into two categories: positive, summarized as good, slightly good, and normal and negative, summarized as bad and slightly bad (Table 4). Interestingly, the number of positive general wellbeing days was significantly improved in the LC-Plasma group compared with the control during T2 and T3.

### 3.5. Safety of LC-Plasma

No severe adverse or adverse events were reported in either group during the intervention period. It is considered that LC-Plasma has no adverse effects in healthy Vietnamese schoolchildren after 8 weeks continuous oral supplementation.

## 4. Discussion

It was reported to be very important to reduce the incidence of infectious disease in children in Vietnam, where these infectious disease are the major causes of morbidity and death in children [4]. It is well known that the influenza virus, rhinovirus, and respiratory syncytial virus play a major role in URID and it is generally acknowledged that the main prophylactic measures against these infectious diseases are vaccinations and everyday hygienic behaviors, such as gargling and hand-washing. These hygienic measures have dramatically improved due to the response towards the COVID-19 pandemic in 2020 and have played a notable role in the marked decrease in influenza, with little to no influenza activity since the start of the pandemic [24].

There is insufficient evidence that antibiotic use reduces the risk of pneumonia in children [25]; however, a previous study reported that children supplemented with probiotics had fewer number of days with URID per person [26]. In view of the economic burden of these infectious diseases and the harmful effects of drug abuse, including antibiotics, infection prevention strategies using probiotics is rational. In addition, both paraprobiotics (heat-killed probiotics) and probiotics retain their immune-modulatory potency beyond their viability [11]. A previous study reported on the prophylactic effects of LC-Plasma on influenza-like illnesses in healthy volunteers during the winter season, and LC-Plasma reduced the cumulative incidence rate of influenza among schoolchildren [17]. Therefore, we focused on schoolchildren and investigated the efficacy of LC-Plasma in reducing the cumulative days absent from school due to infectious disease, including URID, in Vietnam. 

The effect of LC- Plasma on virus infection was verified using virus infected mouse models [13]. It was previously reported that LC-Plasma stimulates murine pDC to produce large quantities of type I IFNs [27]. The general effect of probiotics on the induction of salivary immunoglobulin A levels, bacteriocins, and reuterin, which have antimicrobial activity, is suggested to be a preventive effect on URID [28].

The cumulative absent days and the number of absent students due to GID in this study was much lower than those due to URID, thus we focused on the effects of LC-Plasma on URID due to the limitation of low numbers of absent students due to GID. There was a significant reduction in the number of school absence days due to URID in the LC-Plasma group compared with the control group during T2; however, there was no significant difference between groups during T3. We suggest two possible reasons for the difference between T2 and T3. The first suggestion is that the season changes in northern Vietnam from the end of September to November (T1 to T2), and based on the past (2020) climate data in Ninh Binh province (https://www.worldweatheronline.com/ninh-binh-weather-averages/vn.aspx, accessed on 20 January 2022), the average temperature (25 in T2 to 24 degree Celsius in T3) and humidity drastically decrease (Precipitation in 865.6 mm in T2 to 290.6 mm in T3). During T3 to T4, the season is winter from December onward. It was recently reported that environmental factors, especially temperature and humidity, play an important role in infection rates: affecting host intrinsic, innate, and adaptive immune responses to viral infections in the respiratory tract [29]. The seasonal changes overlap with the seasonal morbidity rates from ILI in Vietnam [4] and infection rates by the common cold virus is considered to increase during T2. The second reason is due to social issues: in Vietnam, the new school year begins from September, and the first school examinations for terms evaluation are set in December, meaning that participants and their guardians are more hesitant about being absent from school even if school students had mild/moderate symptoms. In addition, during T1 through T4, no COVID-19 patients were reported in Ninh Binh province. In future, we have to carry out more detailed clinical study in similar environment for exploring the mechanism of LC-Plasma. The data suggests that LC-Plasma intake could attenuate the physical burden of URID in schoolchildren, resulting in a reduction in the number of absent days per student.

LC-Plasma is reported to have preventive effects on URID, including the common cold and influenza, via enhancement of an IFN-α-mediated response [16]. LC-Plasma intake was predicted to improve mild URID symptoms; however, further detailed studies, including objective clinical data using more symptomatic subjects, should be conducted to evaluate the effects of LC-Plasma intake in high risk areas of infectious disease.

There was a slight decrease in the intensity of general wellbeing score in the LC-Plasma group during T2 and T3; therefore, we analyzed the wellbeing score over an accumulation of days. LC-Plasma significantly improved the number of positive wellbeing days when compared with the control group during T2 and T3. The discrepancy between the two results could be because the sensitivity of the general wellbeing parameter was not strong enough to detect a daily change or because there was a relatively high rate of incidence of asymptomatic and/or upper respiratory disease with very slight symptoms of respiratory virus disease [30], making it difficult to detect an overall condition. In future studies, the parameter for general wellbeing will have to be reconsidered. Recently, Galatini et al. reported on the importance of asymptomatic respiratory virus infections, since asymptomatic infection rates exceeded 70% for most viruses, except influenza and human metapneumovirus, which produced significantly more severe outcomes [30].

Vietnam is working towards eliminating underweight and stunted growth in children and one of the important issues to be considered is nutrition. We surveyed the nutritional status of the participants and compared the change in body weight and height with previous nutritional survey studies in Vietnamese schoolchildren. Our data showed the average body weight gain as 0.43–0.45 kg/8 weeks and height gain as 0.88–0.90 cm/8 weeks. Lien et al. reported body weight gain as 0.34 kg/8 weeks and height gain as 0.87 cm/8 weeks [31] and Hall et al. reported body weight gain as 0.31 kg/8 weeks and height gain as 0.84 cm/8 weeks [32]. We also evaluated underweight, stunting, and wasting using the Z-score for weight/age, height/age, and weight/height/age, respectively. The ratio of underweight in each group was 10.1% in the control group and 9.9% in the LC-Plasma group, the ratio of stunting in each group was 6.6% in the control group and 5.7% in the LC-Plasma group, and the ratio of wasting in each group was 5.0% in the control group and 6.0% in the LC-Plasma group after the 8-week intervention period (data not shown). In 2009, Lien et al. reported the ratio of underweight as 35.4%, stunting as 7.3%, and wasting as 11.3% in a Vietnamese school student study [30]. Our data showed that malnutrition has improved in Vietnam in the last decade.

In conclusion, this is a randomized, placebo-controlled, double-blind, parallel group study of elementary schoolchildren, grades 1 to 3, in northern Vietnam. The intervention was a non-invasive study of LC-Plasma intake. Our results showed that LC-Plasma significantly reduced the cumulative absent days from URID, GID, and URID during the first 4 weeks of intervention and there was a tendency towards a decrease in the cumulative absent days from URID, GID, and URID for the total intervention period. In addition, LC-Plasma reduced the cumulative fever positive days during T2. The cumulative days of positive general wellbeing was significantly improved by LC-Plasma intake during the intervention period. The growth of participants was well maintained, as judged by body weight and height gain, and the malnutrition status was not affected by LC-Plasma. There were no safety concerns for LC-Plasma in this study. In addition, the study product itself is only for produced for clinical trial and not a commercialized product, however, the beverage including LC-Plasma is sold at an affordable price range in Vietnam (0.4 to 0.5 USD/bottle). LC-Plasma is considered to improve the health condition of elementary schoolchildren and reduce school absenteeism through infectious diseases, especially URID; it is an affordable and safe adjunctive nutraceutical option in Vietnam.

## Figures and Tables

**Table 1:** Background information of participants at enrollment.

	Age (Year)	Gender (Male/Female)	Body Weight (kg)	Body Height (cm)
Control	6.86 ± 0.91	237/219	21.7 ± 4.4	119.0 ± 6.8
LC-Plasma	6.78 ± 0.95	218/218	21.5 ± 4.7	118.8 ± 6.6
*p*-value	0.16	0.56	0.22	0.51
	***Z*** **-Score**			
	**Weight/Age**	**Height/Age**	**Weight/Height/Age**	
Control	−0.66 ± 1.16	−0.68 ± 0.87	−0.39 ± 1.23	
LC-Plasma	−0.68 ± 1.27	−0.67 ± 0.90	−0.44 ± 1.31	
*p*-value	0.32	0.75	0.21	

Data are shown as Mean ± SD, except for gender, gender shows the number (male/female). All data were not significantly different between two groups. *Chi*-square test is used for gender analysis, the other were analyzed by Wilcoxon rank sum Test. LC-Plasma represents *Lactococcus lactis* strain Plasma.

**Table 2:** Cumulative school absent days caused by disease (URID/GID).

Absent by	Terms	Group	Cumulative Days		*p*-Value	Odds Ratio (95% CI)
			Not Absent	Absent		
URID/GID	Term 2	Control	12,698	70 **	0.004	0.57 (0.38~0.84)
		LC-Plasma	12,170	38		
	Term 3	Control	12,739	29	0.49	1.19 (0.73~1.95)
		LC-Plasma	12,175	33		
URID	Term 2	Control	12,706	62 **	0.005	0.56 (0.37~0.85)
		LC-Plasma	12,175	33		
	Term 3	Control	12,746	22	0.38	1.28 (0.74~2.24)
		LC-Plasma	12,181	27		
GID	Term 2	Control	12,760	8	0.45	0.65 (0.23~1.89)
		LC-Plasma	12,203	5		
	Term 3	Control	12,761	7	0.84	0.90 (0.32~2.54)
		LC-Plasma	12,203	6		

*Chi*-square test is used for statistical analysis. *p*-value and odds ratio and its 95% confidence interval (CI) are shown. ** represents significant difference between Control and LC-Plasma (*p* < 0.01). URID represents upper respiratory infectious disease, and GID represents gastrointestinal infectious disease.

**Table 3:** Cumulative symptomatic days with URID and GID.

Symptom	Terms	Group	Cumulative Days		*p*-Value	Odds Ratio (95% CI)
			Negative	Positive		
Fever	Term 2	Control	12,660	108 **	0.001	0.58 (0.42~0.79)
		LC-Plasma	12,148	60		
	Term 3	Control	12,728	40	0.93	1.02 (0.66~1.58)
		LC-Plasma	12,169	39		
Cough	Term 2	Control	10,340	2428	0.85	1.01 (0.95~1.07)
		LC-Plasma	9875	2333		
	Term 3	Control	10,196	2572	0.22	1.04 (0.98~1.11)
		LC-Plasma	9673	2535		
Runny nose	Term 2	Control	10,811	1957 ^#^	0.07	0.94 (0.87~1.01)
		LC-Plasma	10,437	1771		
	Term 3	Control	10,688	2080	0.83	1.01 (0.94~1.08)
		LC-Plasma	10,207	2001		
Constipation	Term 2	Control	9705	3063 ^$^	0.03	1.06 (1.01~1.13)
		LC-Plasma	9138	3070		
	Term 3	Control	9798	2970 ^#^	0.050	0.94 (0.89~1.00)
		LC-Plasma	9495	2713		
Diarrhea	Term 2	Control	12,477	263	0.11	0.86 (0.72~1.03)
		LC-Plasma	11,990	218		
	Term 3	Control	12,471	269 **	0.01	0.78 (0.65~0.94)
		LC-Plasma	12,005	203		
Abdominal pain	Term 2	Control	12,399	369	0.56	1.04 (0.90~1.21)
		LC-Plasma	11,840	368		
	Term 3	Control	12,470	298 ^#^	0.08	0.86 (0.73~1.02)
		LC-Plasma	11,962	246		

^#^ *p* < 0.1, ** *p* < 0.01 (LC-Plasma is lower than Control), ^$^ *p* < 0.05 (Control is lower than LC-Plasma). *Chi*-square test is used for statistical analysis. *p*-value and odds ratio and its 95% confidence in-terval (CI) are shown.

**Table 4:** Cumulative days with Positive or Negative general wellbeing condition.

Group	Terms	Cumulative Days of General Wellbeing		*p*-Value	Odds Ratio (95% CI)
		Positive ^1^	Negative ^2^		
Control	Term 2	10,506	2262 *	0.03	0.93 (0.87~0.99)
LC-Plasma		10,175	2033		
Control	Term 3	10,776	1992 *	0.04	0.93 (0.87~0.99)
LC-Plasma		10,416	1792		

^1^ Positive means sum of day with general wellbeing of good, slightly good and normal condition. ^2^ Negative means sum of day with general wellbeing of slightly bad and bad condition. * *p* < 0.05, *Chi*-square test is used for statistical analysis, *p*-value and odds ratio and its 95% confidence interval (CI) are shown.

## Supplementary Materials

The following are available online at https://www.mdpi.com/article/10.3390/nu14030552/s1, Table S1. Change in body weight and height, and Z-score, Table S2. Intensity of Disease score analysis, Table S3. Degree of General wellbeing score analysis.
