# Supplementary material for: Impact of Infectious Disease after Lactococcus lactis Strain Plasma Intake in Vietnamese Schoolchildren: A Randomized, Placebo-Controlled, Double-Blind Study
Source: Nutrients. 2022 Jan 27;14(3):552. doi: 10.3390/nu14030552 (PMC8839753; doi:10.3390/nu14030552)
Supplement: Supplementary file 1 [file nutrients-14-00552-s001.zip › nutrients-1417290-supplementary.pdf]

Table S1. Change in body weight and height, and Z-score

	Body weight	Body height	Z-score		
**End of Term 1**	**(kg)**	**(cm)**	**W/A^{1}**	**H/A^{2}**	**W/H/A^{3}**
**Control**	**22.0 ± 4.5**	**119.4 ± 6.8**	**-0.63 ± 1.14**	**-0.68 ± 0.86**	**-0.34 ± 1.20**
**LC-Plasma**	**21.8 ± 4.8**	**119.3 ± 6.6**	**-0.65 ± 1.23**	**-0.66 ± 0.91**	**-0.39 ± 1.26**
p-value	0.22	0.53	0.21	0.75	0.32
**End of Term 2**	**(kg)**	**(cm)**	**W/A^{1}**	**H/A^{2}**	**W/H/A^{3}**
**Control**	**22.2 ± 4.5**	**119.9 ± 6.8**	**-0.62 ± 1.12**	**-0.67 ± 0.86**	**-0.33 ± 1.17**
**LC-Plasma**	**22.0 ± 4.8**	**119.7 ± 6.6**	**-0.63 ± 1.22**	**-0.66 ± 0.91**	**-0.38 ± 1.24**
p-value	0.26	0.54	0.18	0.83	0.33
**End of Term 3**	**(kg)**	**(cm)**	**W/A**	**H/A**	**W/H/A**
**Control**	**22.4 ± 4.5**	**120.3 ± 6.9**	**-0.61 ± 1.13**	**-0.67 ± 0.86**	**-0.32 ± 1.17**
**LC-Plasma**	**22.3 ± 4.8**	**120.1 ± 6.7**	**-0.62 ± 1.21**	**-0.66 ± 0.91**	**-0.35 ± 1.22**
p-value	0.27	0.51	0.27	0.81	0.35

1; Weight / Age, 2; Height / Age, 3; Weight / Height / Age. Data are shown as Mean ± SD. All data were not significantly different between two groups. Wilcoxon rank sum is used for statistical analysis.

Table S2. Intensity of Disease score analysis

Score	Term	Control	LC-Plasma	*p*-value
**URID symptom**				
**Fever**	**Term 2**	**0.009 ± 0.031**	**0.005 ± 0.017**	0.42
	**Term 3**	**0.003 ± 0.017**	**0.003 ± 0.015**	0.29
**Cough**	**Term 2**	**0.29 ± 0.35**	**0.28 ± 0.34**	0.93
	**Term 3**	**0.29 ± 0.39**	**0.30 ± 0.39**	0.29
**Runny nose**	**Term 2**	**0.21 ± 0.29**	**0.20 ± 0.27**	0.84
	**Term 3**	**0.21 ± 0.33**	**0.21 ± 0.30**	0.49
**GID symptom**				
**Constipation**	**Term 2**	**0.32 ± 0.45**	**0.32 ± 0.42**	0.93
	**Term 3**	**0.30 ± 0.45**	**0.27 ± 0.40**	0.39
**Diarrhea**	**Term 2**	**0.02 ± 0.08**	**0.02 ± 0.07**	0.85
	**Term 3**	**0.02 ± 0.10**	**0.02 ± 0.06**	0.95
**Abdominal**	**Term 2**	**0.04 ± 0.16**	**0.04 ± 0.12**	0.22
**Pain**	**Term 3**	**0.03 ± 0.12**	**0.03 ± 0.08**	0.85

Data are shown as Mean ± SD. All data were not significantly different between two groups. Wilcoxon rank sum test is used for statistical analysis.

Table S3. Degree of General wellbeing score analysis

Score	Terms	Control	LC-Plasma	*p*-value
**General wellbeing**	**Term 2**	**1.44 ± 0.71**	**1.41 ± 0.71**	0.80
	**Term 3**	**1.41 ± 0.73**	**1.38 ± 0.74**	0.68

Data are shown as Mean ± SD. All data were not significantly different between two groups. Wilcoxon rank sum test is used for statistical analysis.
